# Diagnostic Barriers in Children with Immunodeficiencies in Central Asia: A Case-Based Discussion

**DOI:** 10.3390/pediatric13030055

**Published:** 2021-08-07

**Authors:** Zhanna Dauyey, Dimitri Poddighe

**Affiliations:** 1 Department of Medicine, Nazarbayev University School of Medicine, Nur-Sultan 010000, Kazakhstan; Zhanna.Dauyey@nu.edu.kz; 2 Department of Pediatrics, National Research Center for Maternal and Child Health, University Medical Center (UMC), Nur-Sultan 010000, Kazakhstan

**Keywords:** bronchiectasis, pediatric hypogammaglobulinemia, common variable immunodeficiency, Kazakhstan, Central Asia, diagnostic delay

## Abstract

Common variable immunodeficiency (CVID) is a primary immune deficit (PID) mainly characterized by hypogammaglobulinemia. In addition to increased susceptibility to infections and several immune-mediated manifestations, patients with CVID frequently develop bronchiectasis because of recurrent respiratory infections. This occurrence could be more likely if the diagnosis of CVID is delayed, as it often happens in less resourced clinical settings. A 15-year-old female patient was admitted to a tertiary hospital in Kazakhstan for consultation regarding a previous and established diagnosis of bronchiectasis. The clinical history was characterized by recurrent respiratory infections for several years, in addition to the development of a mixed restrictive-obstructive respiratory syndrome. Therefore, she underwent chest computerized tomography, which confirmed the presence of multiple and bilateral bronchiectasis. The clinical discussion on this patient highlighted that serum immunoglobulins were never measured previously and, thus, their assessment was strongly recommended. Based on that, a diagnosis of CVID was finally achieved, and the patient started the appropriate immunoglobulin replacement therapy. To our knowledge, this report is the first English-language publication on CVID and bronchiectasis from Central Asia. Bronchiectasis is currently an important medical problem in developing countries and populations with low socioeconomic status, where the diagnosis of the underlying cystic fibrosis and non-cystic fibrosis comorbidities can be delayed and more difficult than in countries with more accessible health care systems and facilities. This case report emphasized this important clinical issue in Central Asia and should raise the medical attention and awareness of this health problem, in order to improve the diagnostic timing and rate.

## 1. Introduction

Common variable immunodeficiency (CVID) is a primary immune deficit (PID) characterized by profound hypogammaglobulinemia, which predisposes patients to develop recurrent and/or severe infectious diseases affecting several systems, especially the respiratory and gastrointestinal tract. In detail, recurrent respiratory infections (including pneumonia, bronchitis, sinusitis, and otitis) are observed in the majority of CVID patients. In their clinical history, especially if CVID is not timely diagnosed and treated, recurrent infections involving the lower respiratory airways and lungs can cause irreversible structural damages of the lungs (pulmonary sclerosis) and bronchial tree (bronchiectasis), which gradually lead to the development of chronic obstructive and/or restrictive pulmonary syndromes [1,2]. Moreover, the clinical picture of CVID often includes autoimmune manifestations, affecting several organs. As regards the lungs, immune-mediated pathological changes, such as the interstitial lung disease and pulmonary granulomas, can further increase the rate of pulmonary complications and negatively impact on the respiratory function of CVID patients [3,4].

The etiopathogenesis of CVID is very complex and is supposed to involve a multitude of alterations at the molecular and cellular level, which finally result in the development of hypogammaglobulinemia and the related clinical picture (often including immune-mediated and autoimmune manifestations, in addition to the susceptibility to infections), probably with the variable contribution of several and not well-defined environmental factors [3]. Indeed, despite the inclusion of CVID in the PIDs classification, genetic defects/mutations can be currently ascertained in a minority of cases and, importantly, those can be variably inherited (by either autosomal recessive or dominant pattern) and also have a variable penetrance, which is not greater than 30% [5,6]. Accordingly, only 10% to 15% of CVID patients have at least one first-degree relative affected with PID, which can be actually CVID as well as a different antibody related PID, including immunoglobulin A deficiency, IgG subclass deficiency, or others [7]. Moreover, unlike most PIDs, the usual onset age of CVID is not in the first years of life, but its incidence peak is reported during the second and third decades of life [2].

Therefore, the diagnosis of CVID may be quite challenging for general pediatricians due to the relatively late onset (compared to the majority of PIDs) and the heterogeneous clinical picture, including also immune-mediated manifestations, which may mislead the diagnostic work-up and differential diagnosis. Briefly, the diagnosis of CVID is defined by the deficit of at least two antibody isotypes (including necessarily IgG), in addition to the exclusion of other concomitant causes of secondary acquired hypogammaglobulinemia (e.g., chemotherapy and biological therapies) and the presence of a consistent clinical picture (including both infectious and non-infectious diseases, such as autoimmune/immune-mediated manifestations, granulomatous diseases and non-clonal lymphoproliferation). If available, the demonstration of a poor antibody response to vaccines can further support the diagnosis of CVID, whenever any diagnostic concerns should be present [2,8].

All these first considerations may explain how more difficult the diagnosis of CVID could be in developing countries and, in general, in less resourced settings, where the health system organization and the access to specialized medical care are more problematic. In this case report, we present an example of delayed CVID diagnosis in Kazakhstan, despite the implementation and availability of national diagnostic-therapeutic protocols for a variety of clinical conditions, including bronchiectasis. Additionally, this is the first article on pediatric CVID (and, in general, PID) and pediatric bronchiectasis from Central Asia.

## 2. Clinical Case Presentation

Following the onset of acute respiratory symptoms, a 15-year-old female patient was admitted to the hospital for consultation about a previous and established diagnosis of bronchiectasis. Indeed, she reported productive cough with green purulent sputum, mild and inconstant dyspnea, and intermittent fever (up to 38 °C) for 4–5 days before the hospital admission.

Her past personal history was characterized by recurrent episodes of upper and lower respiratory infections, which started several years before. Indeed, the parents reported a number of long-lasting episodes of bronchitis (according to their own words) that required several weeks of antibiotic therapy. In general, recurrent respiratory infections have been reported and, among these, at least 2–3 episodes of acute bronchitis (sometimes complicated with pneumonia) per year. Because of the development of a mixed obstructive-restrictive respiratory syndrome when she was 13 years old, the patient underwent chest computerized tomography, which revealed the presence of multiple and bilateral bronchiectasis, in addition to aspects of pneumosclerosis.

No additional complaints or concerns emerged from her clinical history: in detail, her prenatal course was normal, and she was born full-term, healthy and with length/weight appropriate for gestational age. During the first months of life, the physical growth was regular, and she acquired all the developmental milestones at the appropriate age. However, after the first 2–3 years of life (concomitantly with the appearance and growing number of respiratory infections), the parents reported failure to thrive, which became more and more evident over the time. Indeed, at the present physical examination, her weight and height were <10th percentile (height = 156 cm, weight = 49 kg, pubertal development: Tanner stage 2). No (respiratory) allergy was reported. 

At the hospital admission, except for the delayed growth development and respiratory findings, her physical examination was otherwise unremarkable. As mentioned, the chest examination revealed impaired expansion and decreased vocal fremitus on the right field. Accordingly, the vesicular murmur was not clear and diffusely decreased on the same side. The chest auscultation showed multiple and bilateral respiratory sounds, including crackles during inspiration, and wheezing during expiration. No clubbing or cyanosis was noticed. At the hospital admission, the oxygen saturation was in the normal range as well as the heart (92–96 bpm) and respiratory rates (14–18 rpm). However, the spirometry suggested a mixed pattern of restrictive-obstructive respiratory syndrome. 

The available routine laboratory investigations are displayed in Table 1. The cell blood count showed normal leukocytes and platelets count, but the patient had microcytic anemia (associated to serum ferritin approaching the lower limit of the normal range). As for the inflammatory parameters, the erythrocyte sedimentation rate (ESR) resulted to be normal, but the C-reactive protein (CRP) was moderately increased, supporting the presence of an ongoing acute respiratory infection. The hepatic and renal function was intact. Due to her long-lasting clinical history and the significant prevalence of tuberculosis in the country, the patient’s sputum was analyzed: only *S. aureus* and *S. pneumoniae* were detected, whereas *M. tuberculosis* was absent.

**Figure 1:**
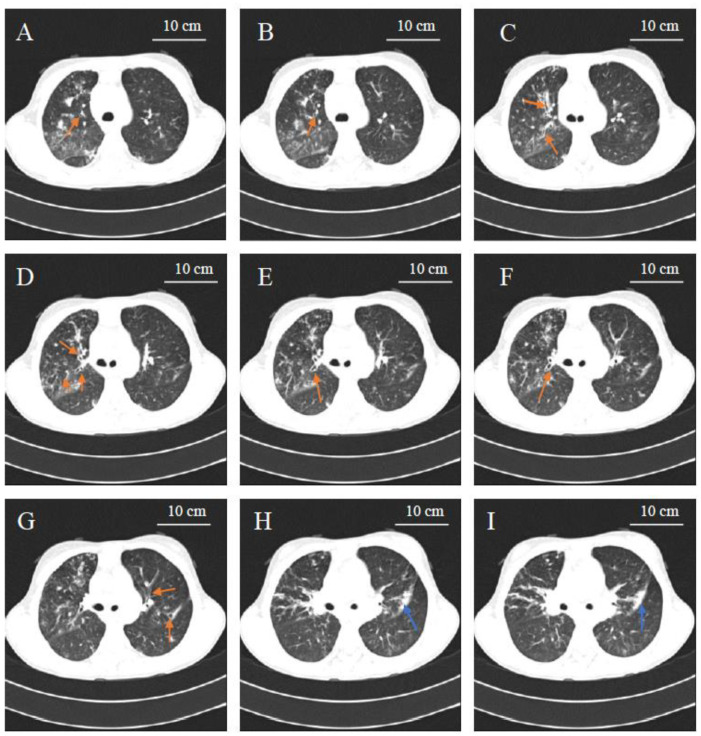
Axial computed tomography (CT) image demonstrating multiple bronchiectasis in the middle lobe of the right lung field (indicated in the **A**–**F** panels with orange arrows), left lower lobe bronchiectasis (highlighted in panel **G** with orange arrows), pulmonary sclerosis in the lower lobe of left lung field (indicated in panels **H** and **I** with blue arrow). CT instrument: Siemens Somatom Sensation 16 EDE 5,6 mSv.

We discussed this clinical case during one active learning session (student’s case conference, MD academic year 3, Nazarbayev University School of Medicine) during the pediatric clerkship, and some important aspects were highlighted. The main point was that, in front of the confirmed radiological picture of bronchiectasis (Figure 1) and the important personal history of recurrent respiratory infections, the patient never received an immunological work-up. Therefore, the simple measurement of serum immunoglobulins (IgA, IgG, IgM and IgE) was recommended, in addition to the sweat test for cystic fibrosis: as reported in Table 2, hypogammaglobulinemia with severe reduction of IgG and total IgA deficiency (without hyper-IgM and hyper-IgE findings) was evidenced, which was consistent with a diagnosis of CVID. Indeed, no absolute cellular deficiency was detected by the following cytofluorimetry analysis of the main lymphocyte subpopulations (CD3+CD8+, CD3+CD4+, CD19+, CD16+CD56+). Accordingly, the patient started the replacement therapy with intra-venous immunoglobulin (IVIG, 0.4 g/kg, every 28 days). After one year since this treatment, this patient showed a significant reduction of the infectious episodes; unfortunately, no precise and confirmed information about the respiratory function and lung radiological picture are available at the moment, since the patient is currently followed at the regional hospital.

## 3. Discussion

To our knowledge, this CVID pediatric case report is the first publication on PIDs and bronchiectasis in Central Asia. Indeed, in this regard no data are currently published on international, peer-reviewed, and English language journals indexed in the most accredited scientific databases (Pubmed, Web of Science, Scopus). Recently, Pilania et al. reviewed the current status of PIDs in Asia: they summarized the epidemiological data from national registries and specialized medical centers for PIDs in Japan, South Korea, China, Hong Kong, Taiwan, South-East Asia, India, Middle East, and Western Asia. However, there was no information on PIDs and their management in Kazakhstan and, generally, in Central Asia [9]. Similarly, as regards bronchiectasis in Central Asia, there are no studies published in international English language journals. Recently, Chandrasekaran et al. reviewed and discussed the geographic variation in the etiology and epidemiology of bronchiectasis worldwide. In addition to cystic fibrosis (which is more prevalent in Caucasian populations than in Asians), bronchiectasis in childhood more frequently is related to primary and secondary immunodeficiency, ciliary dyskinesia, congenital malformations. Unfortunately, this study clearly showed that the available data on bronchiectasis in Asia comes from India, China, and Japan; no data were retrieved from Central Asia [10].

Antibody deficiencies represent the most common group of PIDs: this is attributed to the high prevalence of IgA deficiency (around 1:400), which usually has mild or no clinical manifestations. Indeed, the general prevalence of PIDs worldwide is estimated to be around 1:1200 live births. As for CVID specifically, it affects around 1:25,000 individuals without gender differences, but it accounts for more than 20% of patients included in the national PIDs registries, where only moderate-severe diseases are considered [7,11,12].

The implementation of an official PIDs registry in Central Asia (including Kazakhstan, Kyrgyzstan, Tajikistan, Turkmenistan, and Uzbekistan) or its affiliation with another already existing registry (Russian Federation, for instance) would be desirable and useful. This may help to overcome some important diagnostic issues, including the access to the genetic/molecular diagnostics of PIDs, as highlighted by the present case report.

In terms of the health care system, significant improvements have been made in Kazakhstan in the last few years and the implementation of official national diagnostic-therapeutic protocols for the most prevalent and challenging (acute or chronic) diseases is an important aspect of this process. However, several diagnostic challenges and barriers are still present, as we have evidenced for several other chronic and/or immune-mediated disorders in Kazakhstan and, presumably, in Central Asia [13,14,15,16].

With specific regard to our case report, a specific protocol for the clinical management of patients affected with bronchiectasis is available to guide the diagnostic and therapeutic approach of all physicians/hospitals in the country [17]. Interestingly, hypogammaglobulinemia as an underlying condition leading or associated to bronchiectasis development, was not appropriately considered. Indeed, PIDs (particularly, those associated with hypogammaglobulinemia) are well-recognized causes of (non-cystic fibrosis) bronchiectasis. In detail, patients (including children) with recurrent pneumonia, bronchiectasis or interstitial lung disease should be screened for hypogammaglobulinemia and specifically investigated for CVID [18]. A recent study by Cagdas et al. in children with non-cystic fibrosis bronchiectasis detected PIDs in >40% of cases; importantly, 30% of these bronchiectasis and immune deficient patients were affected with CVID and, in general, an additional 45% of them had a PID with a prevalent antibody deficiency [19]. Indeed, Ho et al. recently reported a large CVID case series (including 624 patients) where 32.3% of participants developed isolated bronchiectasis, and 10.5% showed their coexistence with interstitial lung disease, although they were followed in a tertiary and specialized center in the United States (where a timely diagnosis and appropriate clinical management is presumable, unlike less resourced countries). This study included a population mainly represented by young adults (median age of 25 years and 28 years for males and females, respectively) [20]. Even in a population with a median age at diagnosis falling into the adolescence (like our case report), the prevalence of pulmonary complications and, in detail, bronchiectasis is still prominent. Indeed, those 245 CVID patients included in the retrospective study by Moazzami et al. from Iran, had a median age at the time of diagnosis of 10 years (inter-quartile range 4.0–19.2 years) with a median diagnostic delay of 4 years (inter-quartile range: 1.4–10.0). After a median follow-up period of 3 years (inter-quartile range: 0.8–7), 66 of them (27.2%) had already developed bronchiectasis, which resulted to be the most frequent radiological finding in this cohort of patients [21].

## 4. Conclusions

Currently, bronchiectasis is an important medical problem in developing countries and populations with low resources for the health system, where the diagnosis of the underlying cystic fibrosis and non-cystic fibrosis comorbidities (like PIDs) is often delayed and more difficult than in countries with more accessible health care facilities. This case report clearly emphasized this important clinical issue. In detail, medical awareness should be raised on CVID diagnosis, also in pediatric patients, especially if they are affected with bronchiectasis and/or recurrent respiratory infections.

## Figures and Tables

**Table 1:** General laboratory results at the hospital admission.

Parameter	Result	Units	Reference Range
WBC	9.84	10^9^/L	4.50–13.00
HGB	113	g/L	115.00–150.00
RBC	5.27	10^12^/L	3.80–5.00
MCH	21.40	pg	26.00–34.00
MCHC	30.80	g/dL	31.00–38.00
MCV	69.60	fL	79.00–96.00
PLT	424.00	10^9^/L	150.00–400.00
MPV	9.50	fL	9.00–13.00
Neutrophils	58.80	%	40.50–71.00
Eosinophils	3.40	%	0.50–6.00
Basophils	0.40	%	0.00–0.50
Lymphocytes	29.10	%	18.00–40.00
Monocytes	7.80	%	2.00–10.00
ESR	14.00	mm/h	2.00–15.00
CRP	15.86	mg/dl	0.00–5.00
Cholesterol	3.98	μmol/L	3.21–5.20
Creatinine	44.00	μmol/L	34.00–65.00
Protein	64.70	g/L	64.00–83.00
ALT	15.60	U/L	0.00–44.00
AST	10.10	U/L	0.00–44.00
Ferritin	30.30	μg/L	15.00–150.00

Abbreviations: WBC: white blood cells; HGB: hemoglobin; RBC: red blood cells; MCH: mean corpuscolar hemoglobin; MCHC: mean corpuscolar hemoglobin concentration; MCV: mean corpuscolar volume; PLT: platelets; MPV: mean platelet volume; ESR: erythrocyte sedimentation rate; CRP: c-reactive protein; ALT: alanine aminotransferase; AST: aspartate aminotransferase.

**Table 2:** Serum immunoglobulin levels.

Parameter	Result	Units	Reference Range
IgA	<0.08	g/L	0.47–2.49
IgM	0.1	g/L	0.15–1.88
IgG	0.85	g/L	7.16–17.11
IgE	<1	IU/mL	<100

## Data Availability

The data presented in this study are available on request from the corresponding author. The data are not publicly available due to patient’s confidentiality.
